# Deciphering the Role of Positions 145 and 165 in Fluorescence Lifetime Shortening in the EGFP Variants

**DOI:** 10.3390/biom10111547

**Published:** 2020-11-13

**Authors:** Anastasia V. Mamontova, Aleksander M. Shakhov, Konstantin A. Lukyanov, Alexey M. Bogdanov

**Affiliations:** 1 Center of Life Sciences, Skolkovo Institute of Science and Technology, 121205 Moscow, Russia; sphingozin@gmail.com (A.V.M.); k.lukyanov@skoltech.ru (K.A.L.); 2 Semenov Federal Research Center for Chemical Physics, 119991 Moscow, Russia; physics2007@yandex.ru; 3 Shemyakin-Ovchinnikov Institute of Bioorganic Chemistry, 117997 Moscow, Russia

**Keywords:** fluorescent proteins, GFP, quantum efficiency, oscillator strength, photostability, fluorescence spectroscopy, chromophore, fluorescence lifetime

## Abstract

The bright ultimately short lifetime enhanced emitter (BrUSLEE) green fluorescent protein, which differs from the enhanced green fluorescent protein (EGFP) in three mutations, exhibits an extremely short fluorescence lifetime at a relatively high brightness. An important contribution to shortening the BrUSLEE fluorescence lifetime compared to EGFP is provided by the T65G substitution of chromophore-forming residue and the Y145M mutation touching the chromophore environment. Although the influence of the T65G mutation was studied previously, the role of the 145th position in determining the GFPs physicochemical characteristics remains unclear. In this work, we show that the Y145M substitution, both alone and in combination with the F165Y mutation, does not shorten the fluorescence lifetime of EGFP-derived mutants. Thus, the unlocking of Y145M as an important determinant of lifetime tuning is possible only cooperatively with mutations at position 65. We also show here that the introduction of a T65G substitution into EGFP causes complex photobehavior of the respective mutants in the lifetime domain, namely, the appearance of two fluorescent states with different lifetimes, preserved in any combination with the Y145M and F165Y substitutions.

## 1. Introduction

Green fluorescent protein from *Aequorea victoria* jellyfish (avGFP) and related fluorescent proteins (FPs) from diverse marine creatures possess a unique ability to form a fluorophore group by itself by cyclization and oxidation of three of its own amino acid residues—the chromophore-forming triad (e.g., S65-Y66-G67 in avGFP). Due to this property, FPs can be used as a fully genetically encoded tool for biological imaging [1,2,3]. Numerous investigations demonstrated that even single mutations, especially at the chromophore-forming positions and their intimate neighborhoods, often result in dramatic changes in spectral properties. However, in most cases, there is still no clear understanding of the influence of particular substitutions on FP spectra, which is highly desirable for their rational design. This calls for further detailed mutagenetic studies.

Fluorescence imaging traditionally relies on a signal detection and analysis in a spectral domain, where the wavelength of emitted photons serves as a main qualitative parameter, and the detected light intensity serves as a quantitative one [4]. Multichannel detection, i.e., a simultaneous registration of signals from several probes, represents an important challenge in fluorescence imaging [5,6]. An alternative approach to distinguishing fluorescence signals is their registration in a time domain, where the ultrafast (nanosecond timescale) decay of fluorescence is measured [5,6]. This principle allows the imaging of two to three probes that contrast in their fluorescence lifetimes (FL) in a single spectral channel [7,8,9]. In addition, FL provides a useful quantitative measure of Förster resonance energy transfer (FRET). Although FL is among the characteristics currently used for the selection of promising variants when developing new FPs [10], its structural determinants for these proteins remain poorly understood, which complicates the implementation of the rational approaches to its tuning.

In fluorescent proteins engineering, fluorescence lifetime remains a secondary parameter, rather rigidly tied to the brightness of the protein. When assessing the relationship between fluorescence quantum yield (FQY) and the FL of a fluorophore, researchers usually follow an empirically established, roughly linear correlation between these values [11]. Therefore, in most cases, it is expected that a bright FP has a relatively long FL; conversely, a short FL is a typical sign of unsatisfactory brightness. Noticeably, the fluorescence lifetime of most FPs falls into a narrow range of 2.3–3.5 ns, which generally complicates the composing of the pairs or triads of FP tags optimal for high-contrast time-resolved microscopy. At the same time, the fundamental limits of the FL tuning at a particular FQY (and vice versa) remain largely unexplored.

Recently, we developed bright ultimately short lifetime enhanced emitter (BrUSLEE)—a green fluorescent protein that possesses subnanosecond FL but a rather high FQY of 0.3 and an overall brightness comparable to that of EGFP [12]. Notably, other known FPs with subnanosecond FLs have FQYs of <0.1 [13,14,15]. Thus, BrUSLEE represents a remarkable deviant from the common relationship between FL and FQY, which makes this protein an attractive model to study the structural determinants of these unusual properties.

BrUSLEE differs from the parental EGFP by three mutations: T65G, Y145M, and F165Y. In the parental EGFP [16], the FL value was reported to range from 2.3 to 3.4 ns [9,17,18,19], depending on the excitation regime and external conditions. The T65G mutation of the chromophore-forming residue drastically reduces EGFP brightness and shortens the lifetime to 1.3 ns [12,17,20]. We thus consider this substitution as the primary and key gateway to the FL change in the BrUSLEE relative to EGFP, probably due to a decrease in the average number of hydrogen bonds between the chromophore and its environment, resulting in an increase in chromophore flexibility and changes in its excited-state dynamics [20]. However, FL and brightness are both established through the cooperative action of the chromophore and chromophore-surrounding residues. For instance, in the enhanced yellow fluorescent protein (EYFP), the G65/Y203 combination provides a FL of 3–3.2 ns and a FQY of 0.61 [20]. Introduction of the second mutation, Y145M, leads to further FL reduction down to 800 ps in the T65G/Y145M mutant [12]. These data and the earlier reports [18,21] give reason to consider the Y145M as the second gateway and an important, independent determinant of EGFP FL shortening. The third mutation, F165Y, does not affect the FL but increases the FQY [12].

The comparison of the quantitative characteristics of FPs is complicated due to considerable variations in independently published values. In particular, FL measurement depends on the instrument used, fitting parameters (mean lifetime value of the proteins with multicomponent fluorescence decay vs. single exponential fit), and the protein environment (e.g., purified protein in a cuvette, microscopy of bacteria, or eukaryotic cells). Here, we systematically studied the effects of BrUSLEE-specific substitutions in a detailed side-by-side comparison of their spectral characteristics under identical conditions.

## 2. Results and Discussion

To clarify the role of each substitution and the effects of the combined mutations, we constructed the single EGFP-Y145M and F165Y mutants and double mutants with Y145M/F165Y and T65G/F165Y combinations, and compared their fluorescence characteristics with those of existing EGFP-T65G, T65G/Y145M, and T65G/Y145M/F165Y (BrUSLEE), measuring all proteins under identical conditions.

Absorption and steady-state fluorescence spectra of all the mutants tested appeared to be typical and EGFP-like (Figure S1, Table 1), with some manifestation of a minor neutral chromophore fraction absorbing at 400 nm in Y145M, Y145M/F165Y, and BrUSLEE. Hence, none of the substitutions under investigation participated in the chromophore spectral tuning.

FQYs and extinction coefficients (ECs) of all the proteins lacking T65G mutation were found to be similar to those of EGFP (Table 1). In contrast, all T65G-derived mutants have highly reduced FQY and significantly increased EC.

While the separate effect of the G65 can be interpreted in terms of the increased oscillator strength of the G-Y-G chromophore [20], the combinations of T65G with both Y145M and F165Y probably strengthen this effect (giving an additional increase in EC from 70,000 in EGFP-T65G to ~85,000 M^−1^ cm^−1^ in T65G/Y145M, T65G/F165Y, and BrUSLEE). The introduction of F165Y into EGFP-T65G resulted in a sufficient FQY improvement, thus providing evidence of the possible role of Y165 in an excited-state chromophore stabilization (similar to its functioning in BrUSLEE [12]). Taken together, these observations point to an interplay between an oscillator strength (OS) and chromophore flexibility as the parameters mainly determining the set of the mutants’ practical characteristics (with G65 both increasing OS and destabilizing the chromophore, M145 tuning OS value, and F165 both tuning OS and stabilizing the chromophore).

**Figure 1:**
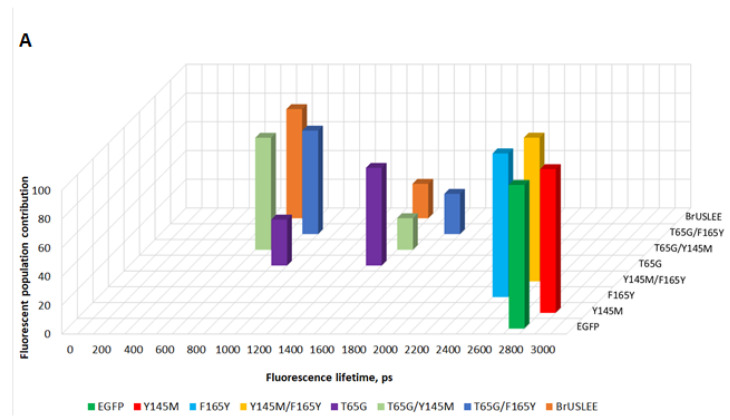

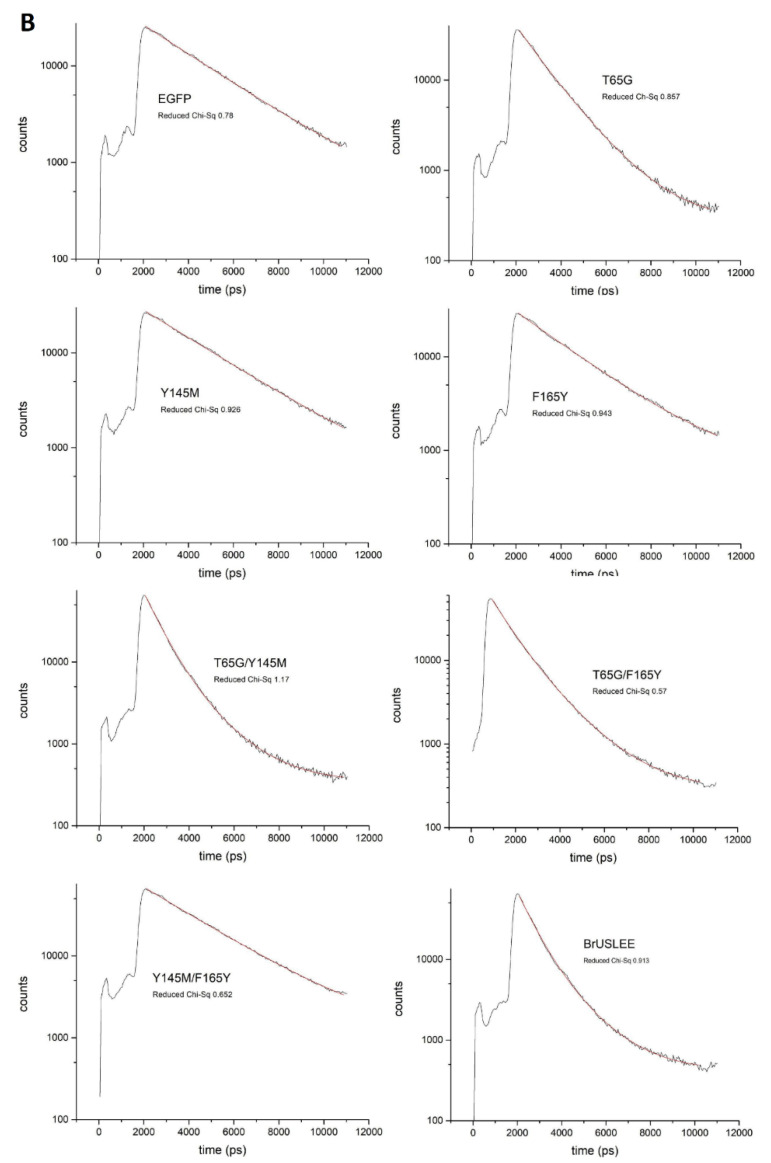
Lifetime measurements in EGFP, BrUSLEE, and the staging mutants. (**A**) A 3D-diagram displaying the lifetime populations found when analyzing fluorescence decays of EGFP; and its mutants at 65, 145, 165 positions, and their combinations. (**B**) Fluorescence decay curves of the purified fluorescent proteins. Experimental decay curves are shown in black, exponential fits are shown in red. Fit accuracy (reduced chi-square) is shown under the name of each protein.

Fluorescence lifetime measurements in eight proteins (Table 2, Figure 1, and Figure S2) revealed important findings that should be considered in tight conjunction with the steady-state data: (i) Y145M and F165Y, as well as their combination, weakly alter EGFP FL, and the fluorescence decay datasets of all mentioned proteins can be fitted with the single-exponential function, thus pointing to the homogeneity in the fluorophores populations within these variants (this is in accord with their impact on EC/FQY values); (ii) the T65G mutation, both alone and in combination with any of 145/165 substitutions, produces at least two fluorescent states.

**Figure 2:**
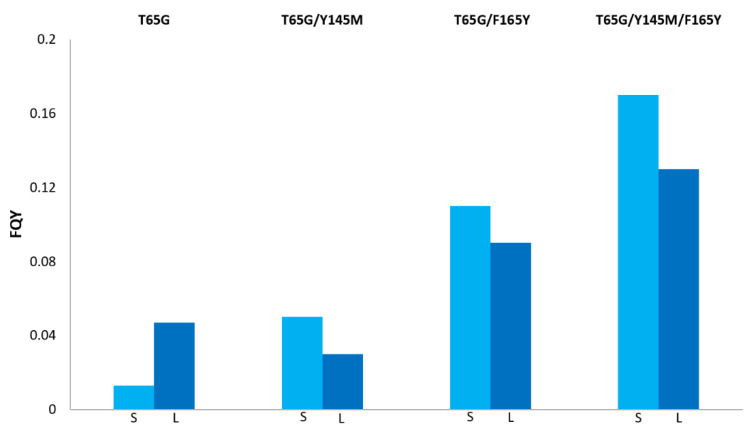
Estimations of FQYs for the separate components observed in biphasic fluorescence decay of the mutants carrying the GYG chromophore. Light blue (S) columns depict the FQY of the short-lived component, deep blue (L) of the long-lived one.

In our experiments, EGFP-T65G, T65G/Y145M, T65G/F165Y, and BrUSLEE showed complex time-resolved fluorescence behavior with at least two distinct reliably detected components: a short one with τ of ~650–900 ps and a long one with τ ~1.5–1.8 ns. We suppose that the T65G mutation represents a single origin of this lifetime divergence, acting probably through the formation of two chromophore states with different degrees of flexibility. This phenomenon might be connected with the T65G-induced changes in the H-bond network within the chromophore environment [20], allowing the ground-state chromophore conformers to have slightly different patterns of H-bonds with the surrounding residues. One cannot also exclude strengthening the impact of the H-bond donor/acceptor residues, surrounding the chromophore, and existing in the alternative rotameric states (e.g., E222 [24]) on the chromophore stabilization; an appearance of the new rotameric variants in residues cannot be excluded, which possess a single spatial conformation in the parental protein. Importantly, the spectral manifestation of the alternative chromophore conformations in the ground state is expected to depend on an efficiency and a rate of interconversions between them. Thus, in the case of a low-energy barrier and fast (µs–ns timescale) conversion, these states would probably be indistinguishable under steady-state spectroscopy. In the EGFP-T65G, the 1.5 ns component dominates (emitting circa 70% photons); Y145M and F165Y mutations added to T65G drastically change this balance in favor of the shorter subnanosecond form. Remarkably, the combination of Y145M and F165Y substitutions did not show a pronounced cooperativity in their impact on the BrUSLEE lifetime characteristics, which is in contrast to their behavior in the spectral domain. One can speculate that the spectrally indistinguishable short and long components might have different intrinsic brightness and a larger EC in the case of the subnanosecond one. Assuming that the total quantum yield is a sum of the components’ FQYs and if the latter are proportional to the product of a lifetime by its contribution (giving A_1_*τ_1_/A_2_*τ_2_ = FQY_1_/FQY_2_ expression), we could estimate the separate FQYs of components observed in biphasic fluorescence decay. The calculations show that: (i) in EGFP-T65G, the FQY of the long-lifetime component is much higher than that of the short one (~0.05 vs. ~0.01); (ii) while the introduction of Y145M does not change a total FQY sufficiently, it provides an increase in the short component’s FQY; (iii) both F165Y alone and a Y145M/F165Y combination added to T65G increase the total FQY, with a stronger enhancement of the short component’s FQY (Figure 2). Importantly, for the EYFP carrying G65/T203 (and its homologs with the same residues pair), the fluorescence lifetimes of 3–3.2 ns and single-exponential decays were reported [20,25].

We should also refine the lifetime values previously reported for the EGFP-T65G, T65G/Y145M, and BrUSLEE [12]. Thus, T65G and BrUSLEE mean lifetimes calculated for in cellulo fluorescence lifetime imaging microscopy (FLIM) experiments were probably originated from the two-component mixture, and the minor components were lost or misinterpreted during the decays unmixing. Similarly, the reported lifetimes (1.3 ns for T65G, 0.8 ns for T65G/Y145M and BrUSLEE) measured in vitro on the isolated proteins reflect mean values rather than the purely single exponential τ, since, due to a domination of the short-lived population, we ignored the minor components and designated them as artifacts. However, the fluorescence behavior of the T65G-derived mutants is characterized by high flexibility, being more sensitive to the experimental conditions (excitation regime, instrument settings, protein solution composition, and even temperature fluctuations) than the conventional EGFP or EYFP (data not shown).

Basic equations of molecular spectroscopy generally describe relations between fluorescence lifetime and brightness. Thus, the Strickler–Berg equation [26] connects the intrinsic fluorescence lifetime of fluorophore with its absorption intensity. Although in many cases the lifetimes predicted by this formula are in good agreement with the experimental values, there are some limitations in its use. One of the key requirements issued to a fluorophore analyzed with the Strickler–Berg equation is that the change in configuration in the excited state cannot be too large. In the original paper, the authors discussed the assumptions granted in the equation and their impact on accurate and detailed predictions. We applied an online tool, calculating fluorophore characteristics using the Strickler–Berg equation [23] to predict the fluorescence lifetimes of the proteins under study and compared these values with the measured ones. As shown in Table 2, for the proteins with monoexponential fluorescence decay, the calculated lifetimes roughly matched the experimental data, prediction accuracy for the mutants with biexponential decay was generally less adequate. One could speculate that for FPs with several fluorescent subpopulations, the Strickler–Berg equation would work better when applied to each fluorescent subpopulation separately. However, since the calculations require the input of an extinction coefficient and a fluorescence quantum yield, which are measured as ensemble values, this separate prediction is likely impossible. Interestingly, the calculated lifetime for the BrUSLEE is similar to that determined experimentally for the short lifetime component. Possibly, this provides additional evidence of the dominance of the short-lived configuration in this protein.

Using the measured rate constants of the excited state radiative (k_r_) and nonradiative (k_nr_) processes, one could directly express the fluorescence lifetime value through the quantum yield, and vice versa. However, it is difficult to quantitatively characterize this ratio for a specific fluorophore, since k_nr_ is the sum of the constants of all possible nonradiative processes (including photoisomerization, charge transfer, singlet-triplet transitions, photochemical reactions, etc.), and the number of such processes and their quantum efficiencies are rarely known in full, especially for such complex fluorophores as proteins.

Finally, we tested whether Y145M and/or F165Y impact the photobleaching rates. Purified, resin bead-immobilized mutant proteins were illuminated with high-intensity blue light using wide-field microscopy (Figure S3, Table S1) to check whether Y145M and/or F165Y impact the photobleaching efficiency. Whereas both an increased photostability and an enhanced resistance to the photoinduced oxidation were earlier documented for most of the T65G-carrying mutants (T65G, T65G/Y145M, and BrUSLEE) [12,20], other mutants have shown either similar (Y145M, T65G/F165Y) or reduced (F165Y, Y145M/F165Y) photostability relative to the EGFP. The photobleaching suppression observed in EGFP-T65G could be connected with its reduced fluorescence lifetime [20]. Y145M appears to have a neutral effect on the photobleaching, and F165Y could play both neutral and negative roles in photostability determination.

## 3. Materials and Methods

### 3.1. Site-Directed Mutagenesis

The EGFP mutants were generated using an overlap-extension PCR technique with the following oligonucleotide set containing the appropriate substitutions: forward 5′-ATGCGGATCCATGGTGAGCAAGGGCGAG, reverse 5′-ATGCAAGCTTTTACTTGTACAGCTCGTC, forward 5′-ACCACCTTCGGCTACGGCCTG, reverse 5′-CAGGCCGTAGCCGAAGGTGGT for EGFP-T65G; forward 5′-GAGTACAACATGAACAGCCAC, reverse 5′-GTGGCTGTTCATGTTGTACTC for EGFP-Y145M; forward 5′-AAGGTGAACTACAAGATCCGC, reverse 5′-GCGGATCTTGTAGTTCACCTT for EGFP-F165Y.

For bacterial expression, a PCR-amplified BamHI/HindIII fragment encoding an FP variant was cloned into the pQE30 vector (Qiagen) with a 6His tag at the N terminus, expressed in the *Escherichia coli* XL1 Blue strain (Invitrogen) and purified using TALON metal-affinity resin (Clontech).

### 3.2. Spectroscopy and Fluorescence Brightness Evaluation

For absorbance and fluorescence excitation-emission spectra measurements, a Cary 100 UV-Vis spectrophotometer and Cary Eclipse fluorescence spectrophotometer (Varian) were used. Fluorescence brightness was evaluated as a product of the molar extinction coefficient by quantum yield multiplication. Measurements on all native proteins were carried out in phosphate-buffered saline (PBS, pH 7.4, Gibco). For molar extinction coefficient determination, we relied on measuring mature chromophore concentration. EGFP and its mutants were alkali-denatured in 1 M NaOH. Under these conditions, a GFP-like chromophore is known to absorb at 447 nm with an extinction coefficient of 44,000 M^−1^ cm^−1^. Based on the absorption of the native and alkali-denatured proteins, molar extinction coefficients for the native states were calculated. For determination of the quantum yield, the areas under the fluorescence emission spectra of the mutants were compared with that of the equally-absorbing EGFP (absolute quantum yield 0.60 [22]).

### 3.3. Fluorescence Lifetime Measurements

Femtosecond laser pulses (80 MHz repetition rate, up to 100 fs, up to 25 nJ per pulse) were generated by a Ti:Sapphire oscillator (Tsunami, Spectra-Physics) pumped by a green Nd:YVO 4 CW laser (532 nm, Millennia Prime 6sJ, Spectra-Physics). Femtosecond pulses were coupled to an inverted optical microscope Olympus IX71 by a Thorlabs FESH0750 dielectric filter mounted at 45° and then focused by an objective lens (40 × 0.75NA, UPlanFLN, Olympus) on a sample, which was placed on a 3-axis stage. The samples were prepared as droplets of the purified fluorescent proteins dissolved in PBS (pH 7.4, GIBCO) applied onto a standard 24 × 24 mm glass cover (Heinz Herenz, Germany). The average laser power was tuned with a polarizing attenuator. A typical laser power coupled to the microscope was about 5 mW. The central wavelength of femtosecond pulses was 980 nm. An SF10 prism compressor was used to compensate for the group velocity dispersion in the objective lens and other optical elements. Fluorescence was excited by two-photon absorption of femtosecond laser pulses, passed back through the objective lens and laser coupling filter, and then directed to the input of an Acton SP300i monochromator with two separate outputs. An EMCCD camera (PI-MAX 2, Princeton Instruments) at the first output was employed for the fluorescence spectra registration. A photomultiplier tube of the time-correlated single photon counting system SPC-730 (Becker & Hickl GmbH) at the second output detected the fluorescence decay kinetics in the 510–530 nm band. Fluorescence decay data were primarily acquired using SPCImage software (Becker & Hickl, Germany) and then exported in ASCII format and analyzed using Origin Pro 9 software (OriginLab, USA).

### 3.4. Photostability Measurements

The resin beads with immobilized proteins were placed in PBS and illuminated with an intense blue light (~1 W/cm^2^) using a fluorescence microscope. Changes in the fluorescence signal in the green channel were monitored during illumination. For wide-field fluorescence microscopy, a Leica AF6000 LX imaging system with Photometrics CoolSNAP HQ CCD camera was used. Fluorescence images were acquired using a 63 × 1.4NA oil-immersion objective and a standard filter set: GFP (excitation BP470/40, emission BP525/50). Photobleaching was monitored in time-lapse imaging in the green channel at low-light intensity combined with 5 s exposures to blue light of maximum intensity (GFP filter set). Images were acquired and quantified using Leica LAS AF software

## 4. Conclusions

Our experiments revealed that the introduction of the T65G mutation to EGFP led to an appearance of two fluorescent forms, both emitting with lifetimes shorter than those in the homogeneous fluorescent population of the parental protein. Both Y145M and F165Y mutations alone, as well as their combination, did not significantly change the fluorescence behavior of the corresponding EGFP variants; however, they distinctly affected the relative contributions of the lifetime components in the T65G-mutants. Hence, while an introduction of M145 and Y165 residues to the environment of the T65-Y66-G67 (TYG) chromophore weakly altered the spectral characteristics, their interaction with the GYG chromophore had sufficient impact on both steady-state (EC and FQY tuning) and time-resolved fluorescence. Although the molecular mechanisms underlying M145 and Y165 influence on the GYG chromophore behavior remain to be established, it is likely that they act in different ways. While the major M145 impact implies tuning of the chromophore electronic configuration (extinction coefficient increase), Y165 mainly works sterically, probably by limiting the chromophore twisting, and thus increasing FQY.

## Figures and Tables

**Table 1:** Fluorescence brightness characteristics of the enhanced green fluorescent protein (EGFP) and its T65G, Y145M, F165Y, T65G/Y145M, T65G/F165Y, Y145M/F165Y, and T65G/Y145M/F165Y mutants.

Fluorescent Protein	λex/λem, nm	EC, M^−1^ cm^−1^	FQY	Relative Brightness, % *	Data Source	
EGFP	489/509	55,000	0.60	100	[22]	
T65G	488/508	70,000	0.06	13	[12,20]	
Y145M	486/508	46,100	0.65	91	this paper	
F165Y	489/509	55,600	0.57	96	this paper	
T65G/Y145M	488/508	84,500	0.08	20	[12]	
T65G/F165Y	488/509	84,000	0.20	51	this paper	
Y145M/F165Y	488/509	56,300	0.72	123	this paper	
T65G/Y145M/F165Y (BrUSLEE)	487/509	86,000	0.30	78	[12]	

* Relative brightness was calculated as a product of the molar extinction coefficient (EC) and the fluorescence quantum yield (FQY) and is reported relative to the brightness of EGFP. For EGFP, the absolute quantum yield is shown; for the mutants, the quantum yields measured relative to the equally-absorbing EGFP are shown.

**Table 2:** Fluorescence lifetimes of EGFP, 65th-, 145th-, and 165th-position mutants and their combinations.

Protein	$\tau_{1},\;\mathrm{ps}$	$A_{1},\;\%$	$\tau_{2},\;\mathrm{ps}$	$A_{2},\;\%$	$predicted\;\tau_{0},\;\mathrm{ns}{\;}^{\#}$
**EGFP**	2830 ± 20	100	-	-	2.08
**T65G**	1530 ± 120	68	890 ± 210	32	0.18
**Y145M**	2935 ± 24	100	-	-	2.57
**F165Y**	2490 ± 15	100	-	-	1.72
**T65G/Y145M**	690 ± 30	78	1575 ± 95	22	0.18
**T65G/F165Y**	905 ± 35	72	1795 ± 95	28	0.51
**Y145M/F165Y**	2595 ± 12	100	-	-	2.16
BrUSLEE *	660 ± 35	76	1500 ± 80	24	0.59

* For the BrUSLEE, a two-component fitting model was chosen. The single-exponential model that we had earlier applied to the BrUSLEE in fluorescence lifetime imaging microscopy (FLIM) experiments here showed unsatisfactory fitting quality. Notably, three-component fitting, producing almost perfect results, could also be used; however, its implementation complicated the BrUSLEE comparison with the homologs, while the contribution of the third component appeared to be negligible. ^#^ Estimated using Strickler–Berg online calculator [23].

## Supplementary Materials

Supplementary materials can be found at https://www.mdpi.com/2218-273X/10/11/1547/s1.
