# Supplementary material for: Deciphering the Role of Positions 145 and 165 in Fluorescence Lifetime Shortening in the EGFP Variants
Source: Biomolecules. 2020 Nov 13;10(11):1547. doi: 10.3390/biom10111547 (PMC7696298; doi:10.3390/biom10111547)
Supplement: Supplementary file 1 [file biomolecules-10-01547-s001.pdf]

biomolecules

Supplemental information

Deciphering the Role of Positions 145 and 165 in Fluorescence Lifetime Shortening in the EGFP Variants

Anastasia V. Mamontova [1], Aleksander M. Shakhov [2], Konstantin A. Lukyanov [1] and Alexey M. Bogdanov [3,*]

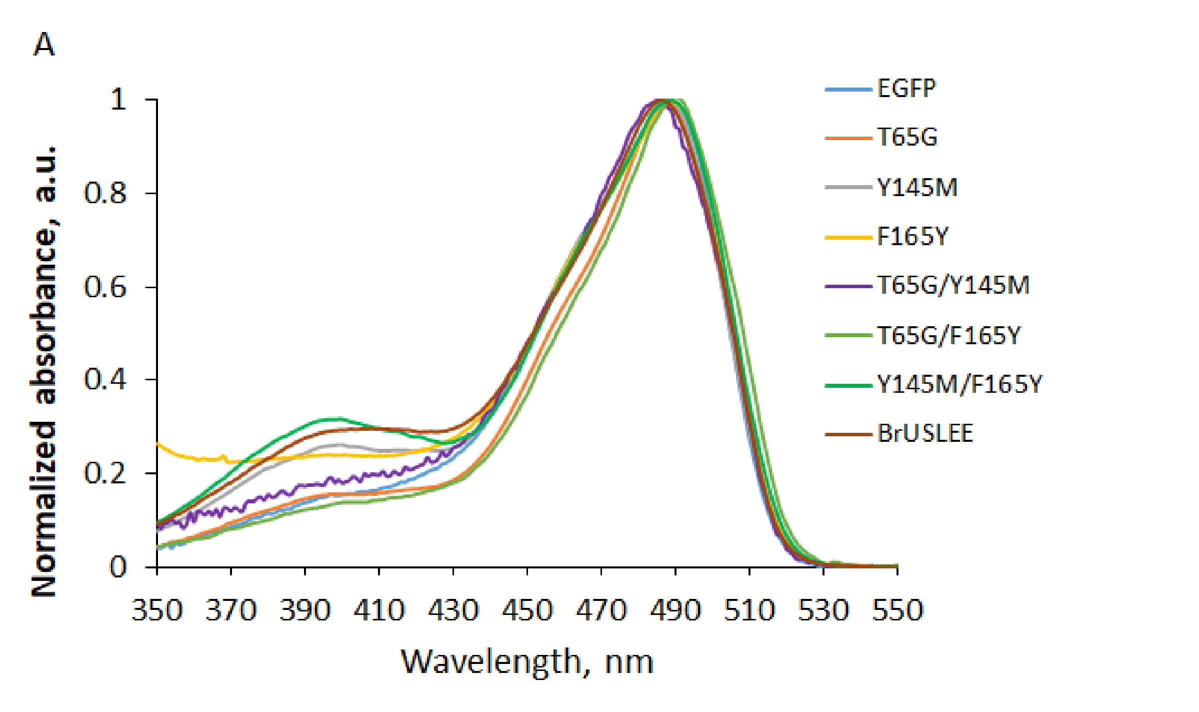

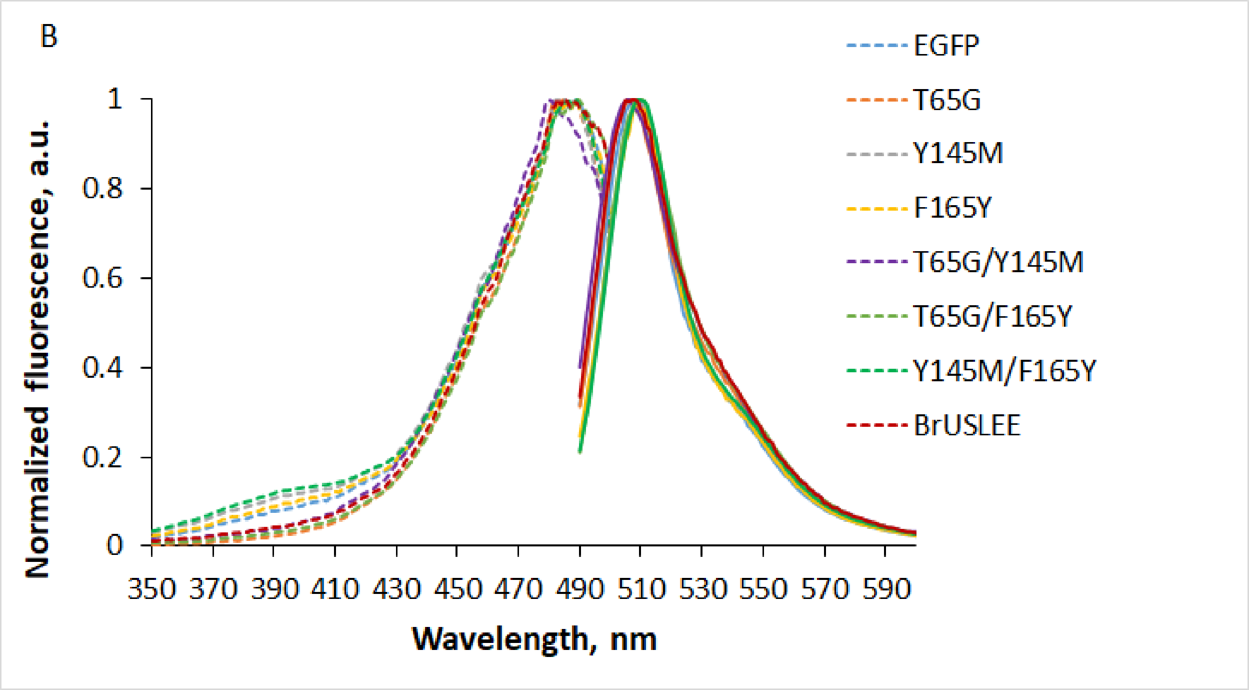

Figure 1. Absorption (**A**) and fluorescence (**B**) spectra of EGFP and mutants. In the fluorescence graph, dashed lines show fluorescence excitation, solid lines – fluorescence emission.

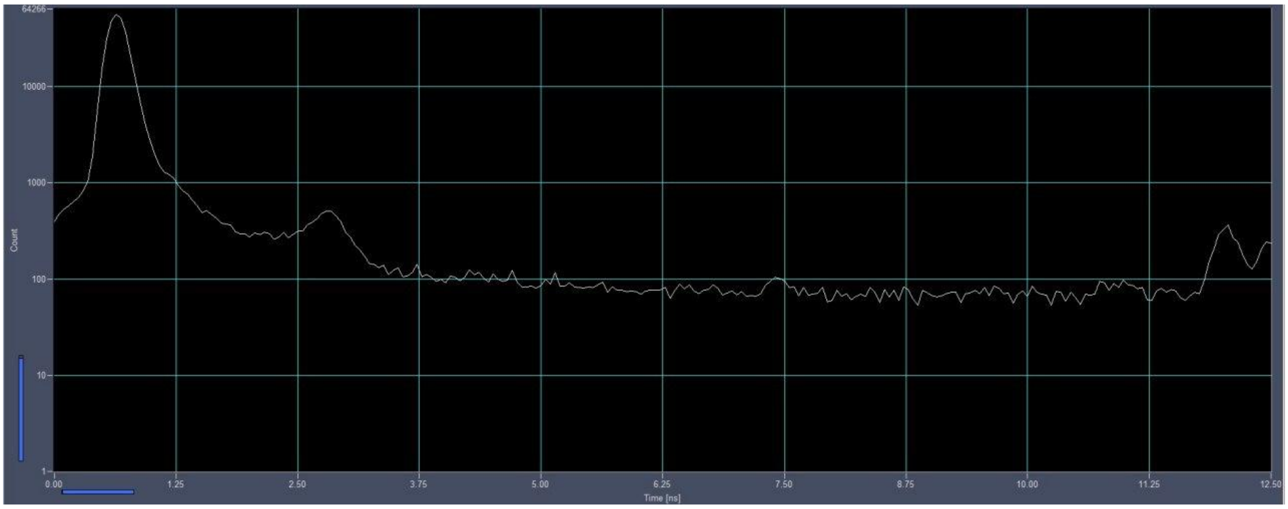

Figure 2. Instrument response function. IRF shape was recorded separately under the same excitation conditions (980 nm) and represented in a screenshot from Becker & Hickl SPCM v. 9.82 data acquisition software. .

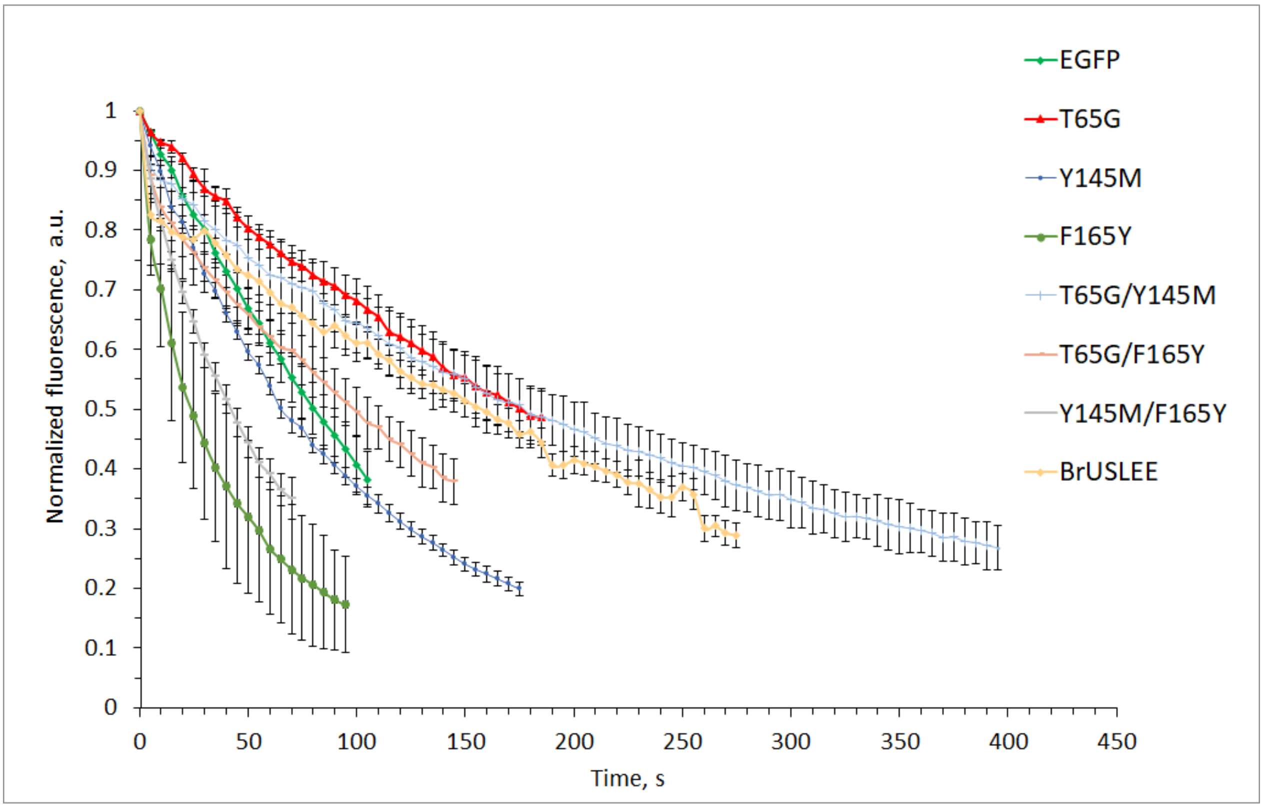

Figure 3. Photobleaching kinetics of the immobilized proteins, EGFP and its mutants, measured in vitro, in PBS. Green fluorescence intensities were background-subtracted and normalized to the maximum values. Standard deviation values (n = 15–20 measurements in a representative experiment out of five independent experiments) are shown.

Table 1. Photostability of EGFP and its mutants *in vitro*.

Fluorescent protein	Photobleaching, s*
EGFP	80±10
T65G	170±25
Y145M	51±5
F165Y	30±6
T65G/Y145M	140±15
T65G/F165Y	65±8
Y145M/F165Y	24±2
T65G/Y145M/F165Y (BrUSLEE)	142±17

*Photobleaching value is represented as the photobleaching half-time for each fluorescent protein, i.e., larger values correspond to the slower photobleaching rate and higher photostability.
